# Endurance Exercise Training Modes to Improve Physical Function in Older Mice: HIIT vs. VWR

**DOI:** 10.1093/geroni/igaa057.3276

**Published:** 2020-12-16

**Authors:** Ted Graber, Chris Byrd, Emily Seguin, Anna Seguin, Alyssa Fennel, Nainika Nadigama

**Affiliations:** East Carolina University, Greenville, North Carolina, United States

## Abstract

With age, we experience a progressive loss of physical function. Exercise is a promising therapy to preserve muscle health and functional aptitude. Animal models are needed to examine the underlying molecular mechanisms at the intersection of aging, exercise, and functional decline. In this study, we compare the efficacy of two types of individualized endurance exercise training in older C57BL/6 mice (26-months old at completion): HIIT (high intensity interval training on a treadmill, n=10) and VWR (voluntary wheel running, n=8). We hypothesized that while both exercises would improve function, HIIT would promote more extensive adaptation. For four months the VWR mice ran 4 days/week and the HIIT group ran 3x/week. We determined function pre/post-training by utilizing our composite scoring system, the Comprehensive Functional Assessment Battery (CFAB). CFAB consists of the following: treadmill running (endurance), rotarod (overall motor function), wheel running (volitional exercise rate/activity), grip strength (fore-limb strength), and inverted cling (overall strength/endurance). EchoMRI determined body composition. After training, we found significant CFAB improvement (repeated measures t-test, p<0.05) in both exercise groups, specifically including: rotarod (+37%, HIIT and VWR); treadmill (+61% VWR; +58% HIIT), grip strength (+20% VWR), body mass (-17% VWR, -10% HIIT), and fat percentage (-44% VWR, -20% HIIT). Contrary to our hypothesis, HIIT did not improve function more than VWR, though we suspect increasing training intensity would improve response. Thus, future studies will need to address defining HIIT dose response and optimal training volume for older mice. We conclude that our models will be useful for future mechanistic investigations.

